# Eye movement desensitization and reprocessing: Exploratory validation study of the potential of a biofeedback digitized approach for burnout therapy optimization

**DOI:** 10.1192/j.eurpsy.2021.319

**Published:** 2021-08-13

**Authors:** R. Maçorano, F. Canais, B. Pereira, K. Drakos, T. Gonçalo, H. Ferreira, M. Parreira

**Affiliations:** 1 Institute For Biophysics And Biomedical Engineering, Faculty of Sciences of the University of Lisbon, Lisboa, Portugal; 2 Faculty Of Medicine, University of Lisbon, Lisboa, Portugal; 3 Institute Of Biomedical Sciences Abel Salazar, University of Porto, Porto, Portugal; 4 Institute Of Biophysics And Biomedical Engineering, Faculty of Sciences of the University of Lisbon, Lisboa, Portugal; 5 Neuropsychology, NeuroGime, Braga, Portugal

**Keywords:** EMDR, burnout, Digital therapy, Biofeedback

## Abstract

**Introduction:**

The Eye Movement Desensitization and Reprocessing (EMDR) therapy has shown to be useful in the treatment of PTSD, general anxiety, stress and burnout. Nonetheless, assessing therapy progress has been limited to subjective appreciations of the patient and therapist, which compromise therapy efficacy, and the continuum of care (clinic and at home) and scalability that digitized approaches can offer.

**Objectives:**

The aim of the present study was to validate the potential of a smartphone-based biofeedback digitized approach for EMDR usage in burnout therapy, as a means to provide quantitative progress assessment and personalized therapy optimization.

**Methods:**

A digitized burnout status assessment app based on Maslach Burnout Inventory was first implemented and tested. Then, an EMDR app was developed by making use of adjustable audiovisual stimuli (e.g. different velocity and horizontal/vertical visual stimuli; and different pitch and left-right surround sound effects) and also of the smartphone’s camera photoplethysmography finger recordings from which heart rate, heart rate variability and breathing rate are derived and used for modulating stimuli (biofeedback). Finally, interviews with several EMDR experts were conducted to assess the potential of the app as a therapeutic adjuvant.

**Results:**

The preliminary interview results showed that the app can be useful for online therapy, to optimize the stimuli presentation, and to quantify the therapy experience and outcomes. The interviews also validated the technical specifications and usability of the tool.

**Conclusions:**

Results so far have shown a promising receptivity and interest from EMDR experts. As such, patient testing is currently on-going.

**Disclosure:**

The work of the present abstract is the basis of the research conducted at the Faculty of Sciences of the University of Lisbon, co-lead with NEVARO, a spin-off company of the same Faculty.

